# Elevation-dependent productivity trade-offs in wild *Elymus sibiricus* populations across the Qinghai–Tibet Plateau zone

**DOI:** 10.3389/fpls.2026.1756195

**Published:** 2026-02-18

**Authors:** Yu lin Niu, Yan Qin

**Affiliations:** 1 Academy of Animal and Veterinary Sciences, Qinghai University, Xining, China; 2 Laboratory for Research and Utilization of Qinghai Tibet Plateau Germplasm Resources, Academy of Animal Science and Veterinary, Qinghai University, Xining, China

**Keywords:** comprehensive evaluation, *Elymus sibiricus*, geographical factors, Qinghai-Tibetan Plateau, SEM

## Abstract

**Background:**

*Elymus sibiricus* is a significant native grass species on the Qinghai-Tibet Plateau, recognized for its ecological and forage value. Elevation serves as a critical environmental factor that affects its growth and development. Therefore, identifying optimal elevations for *E. sibiricus* is essential for its effective selection and utilization.

**Methods:**

In this study, a common garden experimental design was adopted. Six wild *E*. *sibiricus* accessions originating from different elevations were sown in 2022, and a two−year field trial (2023–2024, i.e., the second and third growth years) was conducted in the eastern Qinghai–Tibet Plateau. By observing agronomic traits and measuring forage and seed yields, we employed Mantel tests, K-means clustering, the Technique for Order Preference by Similarity to Ideal Solution (TOPSIS) for comprehensive evaluation, and structural equation modeling to identify optimal elevations for *E. sibiricus* and to determine the key factors influencing yield.

**Results:**

Agronomic traits and yields were significantly improved with extended cultivation duration. Wild *E. sibiricus* accessions were categorized into two groups: one from elevations of 3000–4000 m and the other from above 4000 m.

**Conclusions:**

Forage yield was primarily influenced by population morphology, which was determined by plant height and leaf width, while seed yield was driven by the reproductive architecture, supported by leaf length and effective tillering. Accession 2019−001 achieved the highest comprehensive evaluation score and demonstrated optimal performance, indicating that its elevation of 3147 m may represent a suitable altitudinal range for wild *E. sibiricus*. These findings provide a theoretical foundation and data support for the breeding of *E. sibiricus* in alpine regions.

## 1 Introduction

*Elymus sibiricus* L. is a perennial, caespitose, self-pollinating herbaceous species belong to the Poaceae family and the genus *Elymus* (Zhao et al., 2017). It is widely distributed across the northern temperate zone and high-altitude regions of Eurasia. In China, its natural populations are primarily found in the Qinghai–Tibet Plateau, the Inner Mongolia Plateau, the Yunnan-Guizhou Plateau, and Xinjiang. The species is notably prevalent in alpine meadows and cold meadow-steppe zones of the Qinghai-Tibet Plateau, especially at elevations between 2500 and 4500 m, where it constitutes an important component of natural pasture vegetation (Liu et al., 2022). As a dominant species in the alpine grasslands of the Qinghai-Tibet Plateau (Han et al., 2022), this native grass combines high forage value with essential ecological functions, thereby playing a critical role in regional grassland development and ecological conservation (Zhang et al., 2016). It not only serves as a key species for establishing artificial pastures—thereby grazing pressure on natural rangelands by providing abundant, high-quality forage (Shen et al., 2024; Khan et al., 2024)—but also acts as a pioneer plant in ecological restoration. Its robust root system contributes to soil stabilization, water conservation, rapid vegetation restoration, and the mitigation of grassland degradation, thus maintaining the ecological security of the Qinghai-Tibet Plateau (Zhao et al., 2023). In recent years, with the growing urgency for sustainable development of animal husbandry and ecological conservation in alpine regions, research on breeding and utilization of *E. sibiricus* has been increasingly emphasized. Efforts have predominantly focused on germplasm collection, evaluation (Li et al., 2025), and studies of adaptation mechanisms (Xiong et al., 2024). However, a systematic investigation into its geographical distribution and the key agronomic traits of different ecotypes remains lacking, which hinders subsequent germplasm collection and evaluation of *E. sibiricus*.

The Qinghai-Tibet Plateau, often referred to as the “Third Pole,” is home to flora characterized by short growing seasons, which are primarily influenced by its environmental conditions (Wang et al., 2022a). Elevation serves as a critical factor affecting plant growth. As elevation increases, significant changes occur, including a decline in temperature, heightened UV radiation, decreased atmospheric pressure, altered concentrations of oxygen and CO_2_, limited soil nutrient availability, and increased wind speed (Buckley et al., 2013). Previous research has documented specific adaptations; for example, Pallavi Sati noted that plants at high elevations frequently develop thicker and smaller leaves, along with modified stomatal density, to reduce water loss and withstand intense UV radiation (Sati et al., 2024). Additionally, some species adjust their root and stem growth patterns to better adapt to soil conditions and light availability (Choudhary et al., 2020). A study on Sorghum in the Potwar Plateau of Pakistan revealed that plants at lower elevations exhibited greater height, leaf area, stem length, and fresh weight, indicating more favorable growth conditions at these altitudes (Irshad et al., 2024). In contrast, plants at higher elevations often exhibit slow growth and dwarfism as adaptations to their harsh environments (Zumyil and Antip, 2024). Significant variations in morphological traits and aboveground biomass allocation along an altitudinal gradient have been observed for *Chimonobambusa angustifolia* in the Wolong Nature Reserve (Pan et al., 2009). Additionally, tobacco (*Nicotiana tabacum*) exhibited optimal growth at 2000 m, suggesting species-specific altitudinal preferences (Li et al., 2018). Consequently, different plant species thrive within distinct altitudinal ranges (Rowe and Speck, 2005). Identifying suitable elevations for *E. sibiricus* is critical for its selective breeding. However, the optimal altitudinal range for *E. sibiricus* on the Qinghai-Tibet Plateau remains unclear. The processes and mechanisms by which elevation and growing years affect its yield are not well understood, which hinders breeding progress and highlights the urgent need for targeted research.

This study examined six wild *E. sibiricus* accessions collected from various elevations and cultivated in the eastern Qinghai-Tibet Plateau (Xihai Town, Haibei Prefecture, Qinghai Province). We employed piecewise structural equation modeling (SEM) to analyze the direct and indirect effects of elevation, year, and their interaction on forage and seed yields, including the corresponding path coefficients. A comprehensive evaluation of traits measured over two consecutive years was performed to identify optimal elevational gradients for *E. sibiricus* growth, thereby facilitating its selective breeding for the Qinghai-Tibet Plateau.

## 2 Materials and methods

### 2.1 Experimental site description

**Figure 1:**
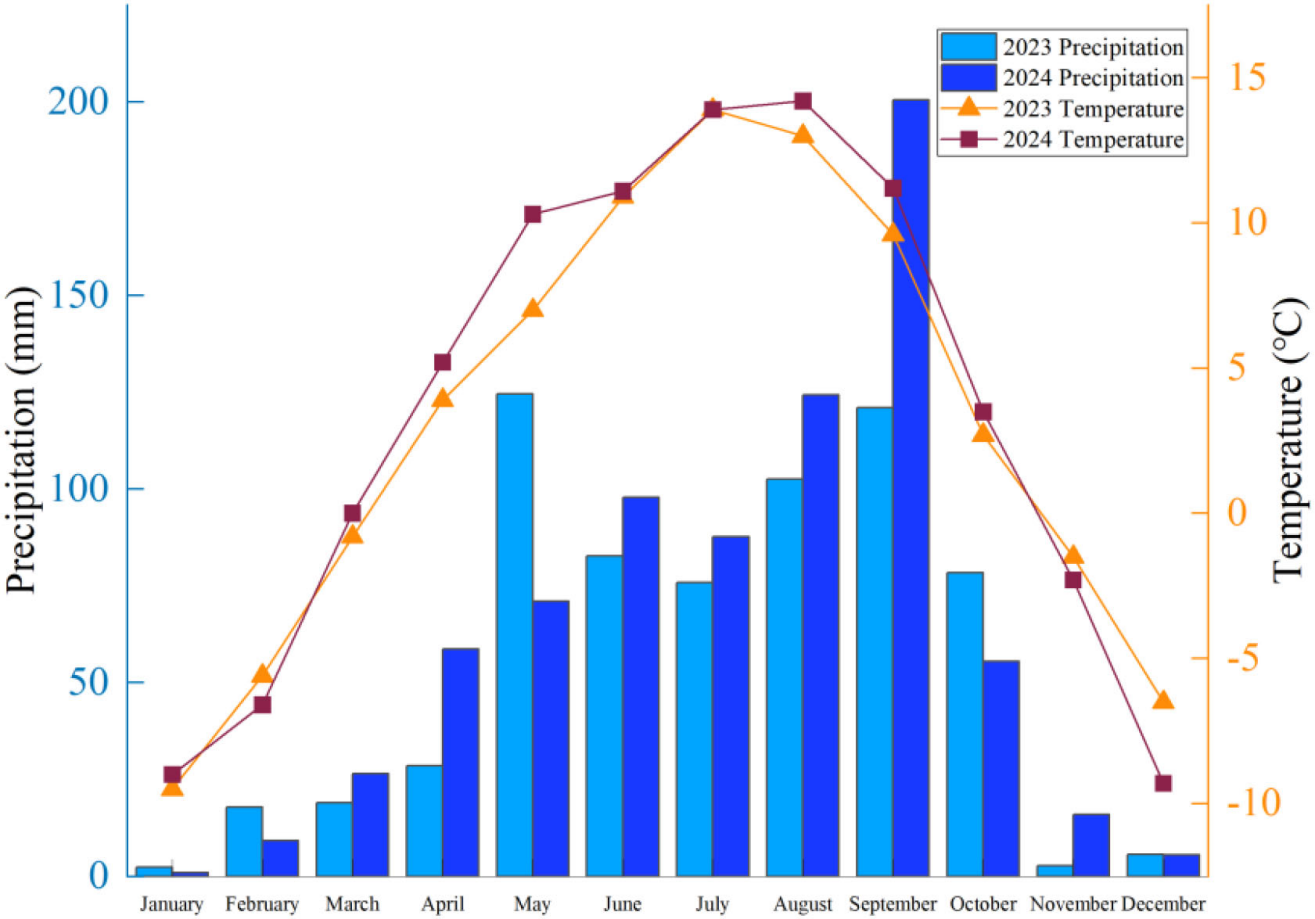
Monthly mean temperature and precipitation at the experimental site from January 2023 to December 2024.

The field trial was conducted at the Forage Reproduction Base in Xihai Town, Haiyan County, Haibei Tibetan Autonomous Prefecture, Qinghai Province (36°59’36” N, 100°52’48” E). This region is characterized by a continental plateau climate and is situated at an elevation of 3,156 m. The mean annual temperature is approximately 0.5 °C, while the mean annual precipitation totals about 369.5 mm, predominantly occurring between July and September. The diurnal temperature variation is significant. The average annual wind speed is 3.3 m/s, and the area enjoys abundant sunshine, receiving approximately 2,970 hours of sunlight annually. The frost-free period extends for about 92 days. Throughout 2023, the mean monthly temperature and precipitation were 3.1 °C and 55.13 mm, respectively (**Figure 1**). In 2024, the corresponding values throughout the year were 3.5 °C and 62.8 mm. The meteorological data were sourced from the China National Meteorological Science Data Center. The soil is classified as Chestnut soil, exhibiting a pH of 8.31. The soil organic carbon content measures 21.15 g/kg, total nitrogen is 3.06 g/kg, and total phosphorus is 0.27 g/kg.

### 2.2 Materials and experimental design

**Figure 2:**
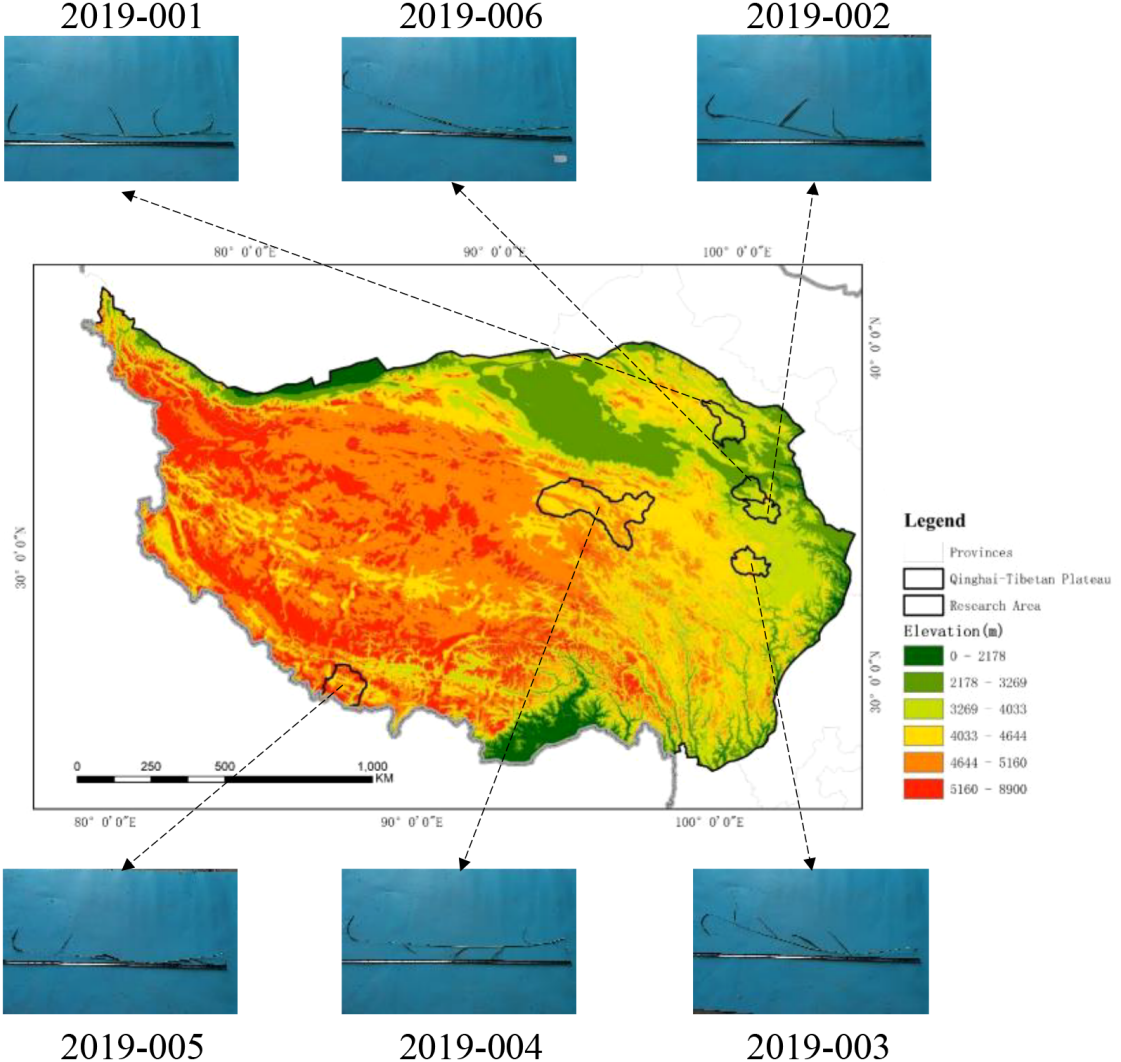
Spatial distribution of experimental materials.

The experimental materials were obtained from the Qinghai-Tibet Plateau (**Figure 2**; **Table 1**). This experiment was conducted using a common garden design, in which soil conditions, water and fertilizer inputs, and management practices were strictly controlled to minimize interference from local environmental factors. As a result, the observed differences in agronomic traits and yield among the accessions can be primarily attributed to their genetic background and the adaptive effects associated with their original elevations. The experimental site was prepared by deep plowing and leveling, followed by the removal of surface stones and weed residues. A randomized complete block design (RCBD) was employed, consisting of five replications. Sowing occurred in mid-May 2022 using the hill-seeding method, with each hill planted with 4–5 seeds at a depth of 3–4 cm. Both plant and row spacing were set at 50 cm. Each plot measured 3 m × 5 m and was surrounded by a 1 m wide buffer row, with a 1 m wide working path maintained between adjacent plots. Diammonium phosphate (DAP) was applied as a basal fertilizer at a rate of 75 kg·ha^-1^. Following seedling emergence, thinning was conducted to ensure that only a single healthy seedling, free of visible pests or diseases, remained per hill. The plants were maintained under natural growth conditions throughout the trial period, without artificial irrigation, topdressing, or grazing. Weed control was performed three times in 2022 and four times annually in both 2023 and 2024.

**Table 1:** Sources of experimental materials.

Germplasm number	Variety name	Collection places	Elevation	Microhabitat
2019-001	*E. sibiricus*	Haibei Tibetan Autonomous Prefecture, Qinghai Province, China	3147 m	Roadside
2019-002	*E. sibiricus*	Huangnan Tibetan Autonomous Prefecture, Qinghai Province, China	3714 m	Roadside
2019-003	*E. sibiricus*	Guoluo Tibetan Autonomous Prefecture, Qinghai Province, China	4017 m	Roadside
2019-004	*E. sibiricus*	Yushu Tibetan Autonomous Prefecture, Qinghai Province, China	4657 m	Roadside
2019-005	*E. sibiricus*	Xigazê City Tibet Autonomous Region, China	4325 m	Roadside
2019-006	*E. sibiricus*	Hainan Tibetan Autonomous Prefecture, Qinghai Province, China	3428 m	Roadside

### 2.3 Sampling methods and index measurement

Systematic sampling was conducted during the full blooming stage of *E. sibiricus* in 2023 (the second growth year) and 2024 (the third growth year). Five uniformly growing plants, exhibiting no evident disease or pest symptoms, were selected from each experimental plot using a systematic sampling method. A calibrated steel tape was employed to measure the vertical distance from the base to the highest growth point, recorded as absolute plant height, as well as the length and width of the flag leaf. Stem diameter was measured with a vernier caliper, and the total number of tillers was recorded simultaneously. Upon completion of the morphological measurements, the plants were cut at ground level. Fresh biomass was determined immediately after removing impurities such as attached soil, litter, and other plant species. The samples were then sealed and transported to the laboratory, where they were fixed at 105 °C for 30 minutes and subsequently dried at a constant temperature of 75 °C until a constant weight was achieved to ascertain dry matter content. At the full ripening stage (late August for both years), seeds were manually collected from individual plants and weighed.

### 2.4 Data analysis

Data were initially organized using Microsoft Excel 2016. Statistical analyses were conducted with SPSS 27.0 (Chicago, IL, USA). The normality of the measured traits—including plant height, leaf length, leaf width, stem diameter, effective tillers, total tiller number, forage yield, and seed yield across different elevations of *E. sibiricus*—was assessed using the Kolmogorov-Smirnov test, along with an evaluation of the homogeneity of variance. A one-way analysis of variance (ANOVA), followed by the least significant difference (LSD) multiple comparisons test (α = 0.05), was performed. To examine trait variations among different germplasm resources, a two-way ANOVA was employed. Data analysis and visualization were performed using R version 4.4.1. K-means clustering analysis was executed using the factoextra and ggplot2 packages in R to identify key phenotypic characteristics. A Technique for Order Preference by Similarity to Ideal Solution (TOPSIS) multi-criteria decision-making model was implemented via the plyr package to comprehensively evaluate agronomic traits, as well as individual plant forage and seed production performance. Mantel tests were utilized to analyze the correlation strength between agronomic traits and production performance indicators. A structural equation model (SEM) was constructed using the piecewise SEM package to quantify the direct and indirect effects, along with path coefficients, of various factors on forage and seed yield, thereby elucidating the regulatory pathways underlying yield formation. Standardized effect values were visualized and finalized using Origin 2022. The Technique for Order Preference by Similarity to Ideal Solution (TOPSIS) is an intuitive and practical multi-criteria decision-making method. It ranks alternatives by calculating the distance between each alternative and the fictitious “ideal best solution” and “ideal worst solution,” and then derives the relative closeness to the ideal solution for ranking (Wang et al., 2022b). Structural Equation Modeling (SEM) is a multivariate statistical analysis method that integrates factor analysis and path analysis. It is primarily used to test whether the proposed theoretical model—which hypothesizes relationships among observed variables and latent variables, as well as among latent variables themselves—fits the data well (Cheung, 2018).

## 3 Results

### 3.1 Effects of elevation on agronomic traits and yield of wild *E. sibiricus*

**Figure 3:**
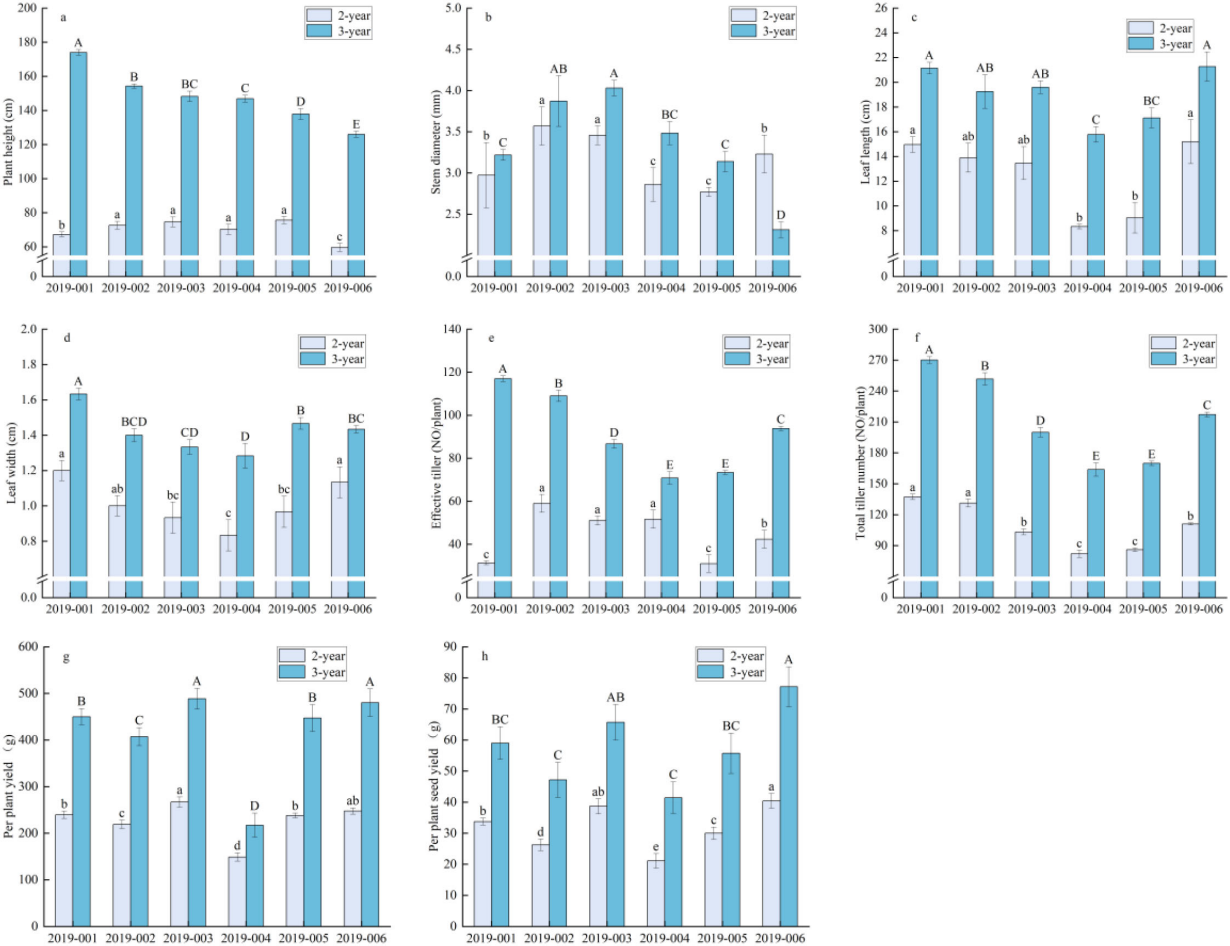
Agronomic traits and yield performance of *E. sibiricus.***(a)** Plant height, **(b)** Stem diameter, **(c)** Leaf length, **(d)** Leaf width, **(e)** Effective tiller number, **(f)** Total tiller number, **(g)** Per plant yield, **(h)** Per plant seed yield. The capital letters indicate significant differences in second growth year germplasm resources (P < 0.05), the lowercase letters denote significant differences in third growth year germplasm resources (P < 0.05); error bars in the figure represent standard errors.

This study examined the agronomic traits and yield of *E. sibiricus* during its second and third years. In the second year, accession 2019–005 exhibited the tallest plant height at 75.67 cm, significantly surpassing all other accessions (*P* < 0.05; **Figure 3a**). Accession 2019–002 displayed the largest stem diameter at 3.57 mm and the highest number of effective tillers, with 59 tillers per plant, demonstrating significant superiority over 2019-001 (*P* < 0.05; **Figure 3b**). The longest leaf length, measuring 45.2 cm, and the highest seed yield of 40.41 g were recorded for 2019-006, both significantly exceeding those of 2019-002 (*P* < 0.05; **Figures 3c, h**). Accession 2019–001 exhibited the greatest leaf width at 1.2 cm and the highest total tiller number, with 137 tillers per plant, significantly outperforming 2019-003 (*P* < 0.05; **Figures 3d, f**). Accession 2019–003 produced the highest forage yield at 266.85 g, significantly surpassing all other accessions (*P* < 0.05; **Figure 3g**). In the third year, accession 2019–001 exhibited superior performance in plant height (174.02 cm), leaf width (1.63 cm), number of effective tillers (117 tillers per plant), and total tiller number (270 tillers per plant), all of which were significantly greater than those of the other accessions (*P* < 0.05; **Figures 3a, d–f**). Accession 2019–003 achieved the largest stem diameter at 4.03 mm and the highest forage yield at 488.57 g, both significantly exceeding those of 2019-001 (*P* < 0.05; **Figures 3b, g**). The longest leaf length (21.26 cm) and the highest seed yield (77.14 g) were recorded in 2019-006, significantly greater than those of 2019-004 (*P* < 0.05; **Figures 3c, h**).

The results of the two-way ANOVA demonstrated that both elevation and plant year exerted highly significant main effects (*P* < 0.01) on plant height, leaf length, leaf width, number of effective tillers, total tiller number, forage yield, and seed yield. Stem diameter was significantly affected solely by the elevation factor. The interaction between elevation and age was highly significant (*P* < 0.01) for plant height, stem diameter, number of effective tillers, and total tiller number. In contrast, no significant interaction effects were detected for leaf length, leaf width, forage yield, or seed yield (**Table 2**).

**Table 2:** Two-way ANOVA of the effects of elevation and years on phenotypic traits and yield in *E. sibiricus*.

Agronomic traits	Treatments	F	*P*
Plant height	Elevation	21.15	<0.01
	Years	2239.75	<0.01
	Elevation×Years	15.32	<0.01
Stem diameter	Elevation	6.89	<0.01
	Years	1.46	0.23
	Elevation×Years	3.63	<0.01
Leaf length	Elevation	8.36	<0.01
	Years	79.94	<0.01
	Elevation×Years	0.32	0.90
Leaf width	Elevation	9.40	<0.01
	Years	157.45	<0.01
	Elevation×Years	0.69	0.64
Effective tiller	Elevation	23.06	<0.01
	Years	657.15	<0.01
	Elevation×Years	23.88	<0.01
Total tillers number	Elevation	93.60	<0.01
	Years	1396.40	<0.01
	Elevation×Years	8.90	<0.01
Per plant yield	Elevation	5.05	<0.01
	Years	58.14	<0.01
	Elevation×Years	1.01	0.42
Per plant seed yield	Elevation	8.08	<0.01
	Years	81.19	<0.01
	Elevation×Years	0.70	0.62

*P*>0.05 indicates no significant difference; *P* < 0.05 indicates significant difference; *P* < 0.01 indicates highly significant difference.

### 3.2 Correlation between yield and agronomic traits in wild *E. sibiricus*

**Figure 4:**
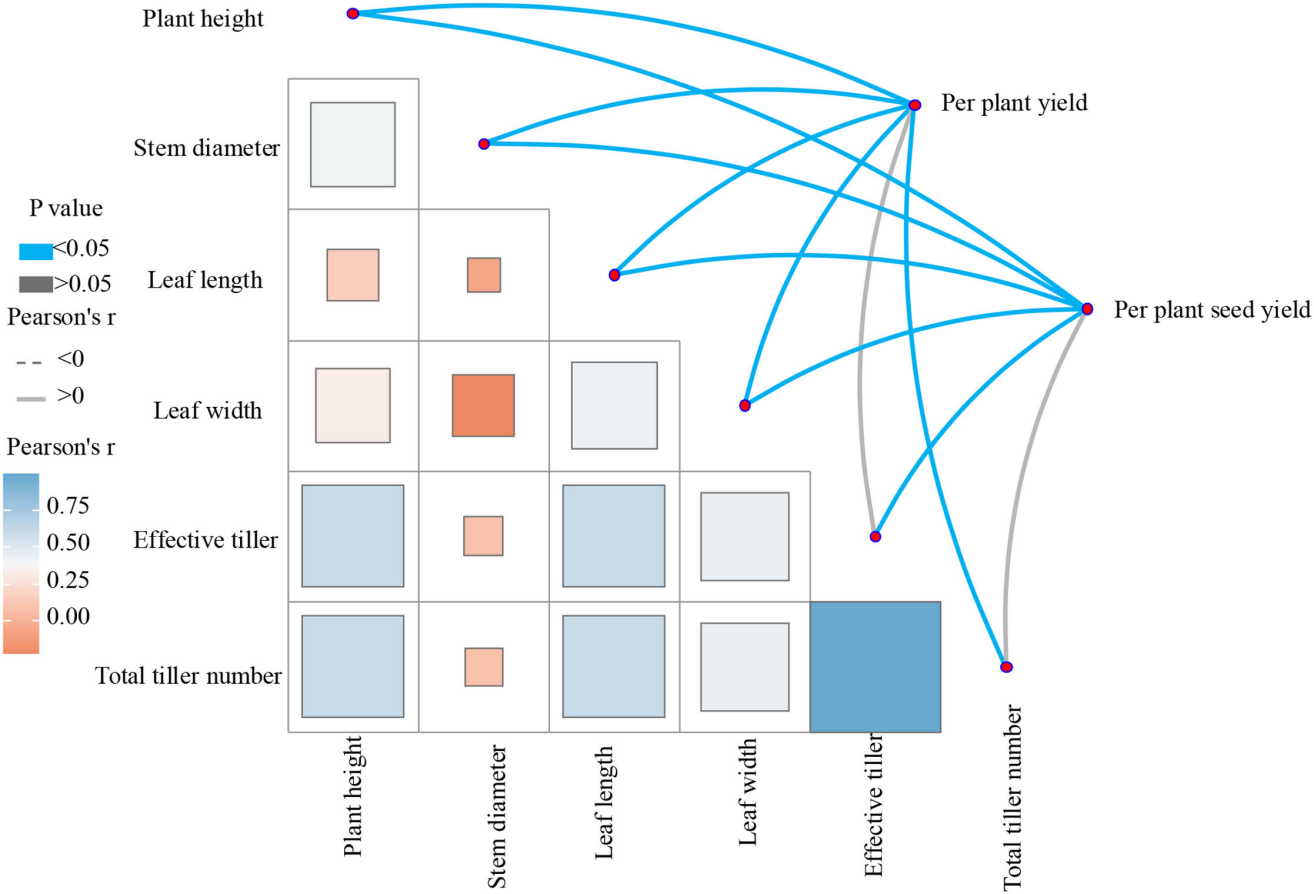
Mantel analysis of correlations between yield and agronomic traits in *E. sibiricus*.

The Mantel test analysis revealed significant positive correlations between forage yield per plant and several morphological traits, including plant height, stem diameter, leaf length, leaf width, and total tiller number (**Figure 4**). Additionally, seed yield per plant exhibited significant positive correlations with plant height, stem diameter, leaf length, leaf width, and effective tiller number. A notable positive correlation was also identified between effective tiller number and total tiller number. Moreover, both effective tiller number and total tiller number demonstrated significant positive correlations with plant height and leaf length.

### 3.3 TOPSIS-based comprehensive evaluation of wild *E. sibiricus* from different elevations

**Figure 5:**
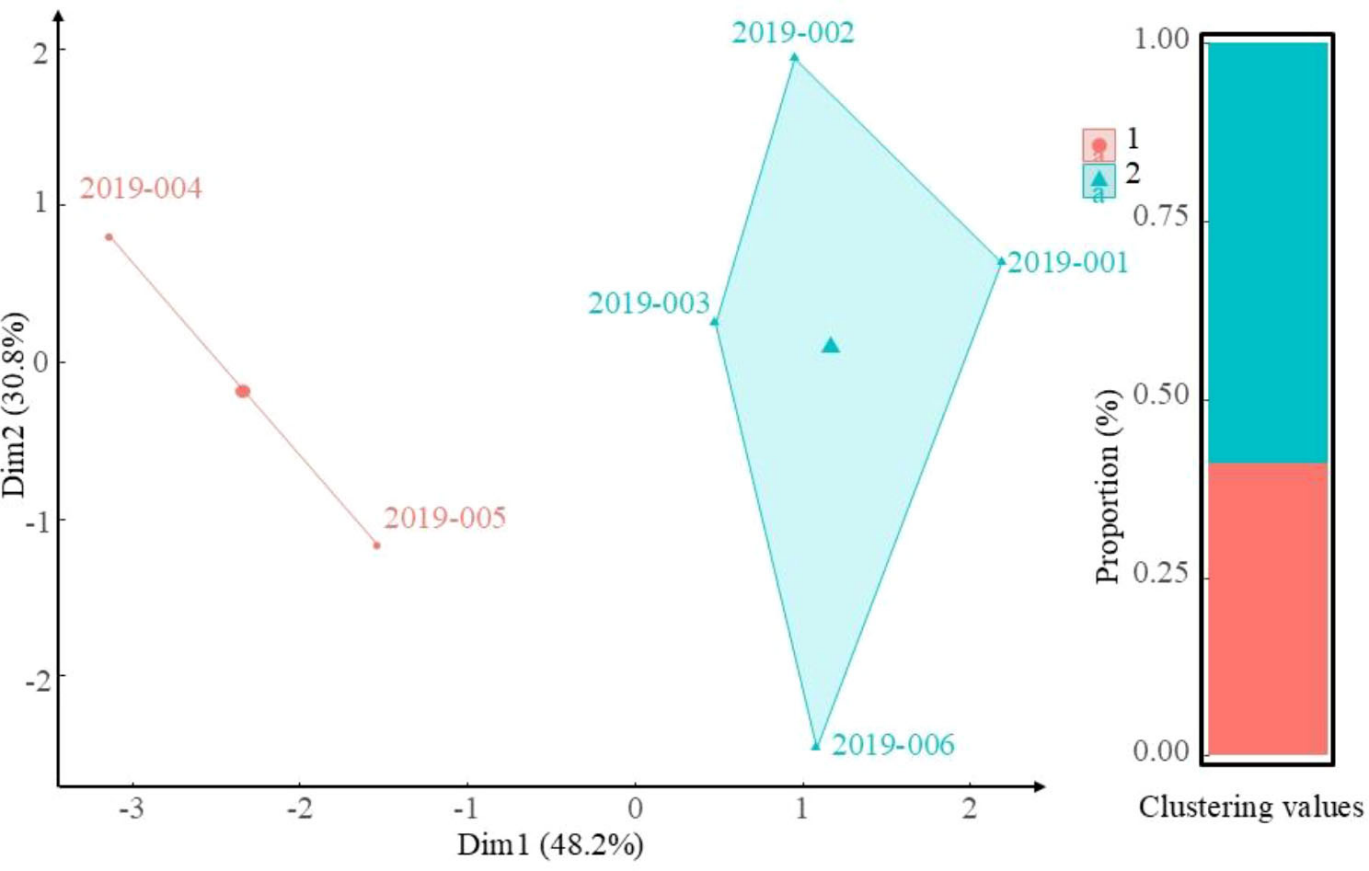
K-means cluster analysis of key traits in *E. sibiricus* across an elevational gradient.

A comprehensive multi-trait evaluation was performed on six wild *E. sibiricus* accessions sourced from varying elevations. K-means clustering analysis, based on key agronomic traits (**Figure 5**), indicated that these accessions could be categorized into two distinct clusters. The first cluster, which included accessions 2019-001, 2019-002, 2019-003, and 2019-006, represented 59% of the total and exhibited superior phenotypic performance. In terms of elevational distribution, Cluster I comprised accessions from elevations of 3000 m to 4000 m, whereas Cluster II contained accessions from elevations exceeding 4000 m.

**Figure 6:**
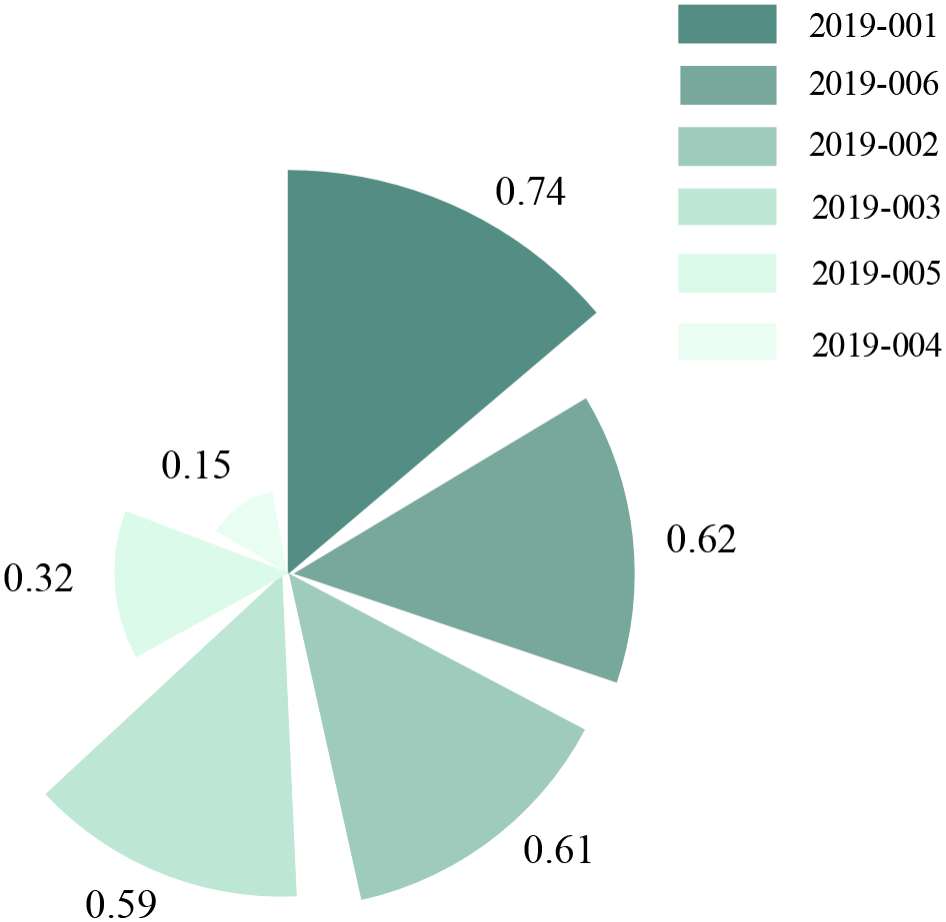
Technique for order preference by similarity to ideal solution comprehensive evaluation of *E. sibiricus* across an altitudinal gradient.

To identify appropriate elevational gradients for wild *E. sibiricus*, a comprehensive evaluation model was developed utilizing a multi-criteria decision-making approach known as TOPSIS. This model incorporated several key parameters, including plant height, stem diameter, leaf length, leaf width, number of effective tillers, total tiller number, forage yield, and seed yield (**Figure 6**). The evaluation results revealed a descending order of similarity scores: 2019-001 > 2019-006 > 2019-002 > 2019-003 > 2019-005 > 2019-004. Accession 2019–001 achieved the highest similarity score of 0.74, indicating superior overall performance, whereas accession 2019–004 recorded the lowest score of 0.15. Therefore, the elevational region from which 2019–001 was collected (3147 m) may represent the optimal elevational gradient for wild *E. sibiricus*.

### 3.4 Pathways of elevational effects on forage and seed yield in wild *E. sibiricus*

**Figure 7:**
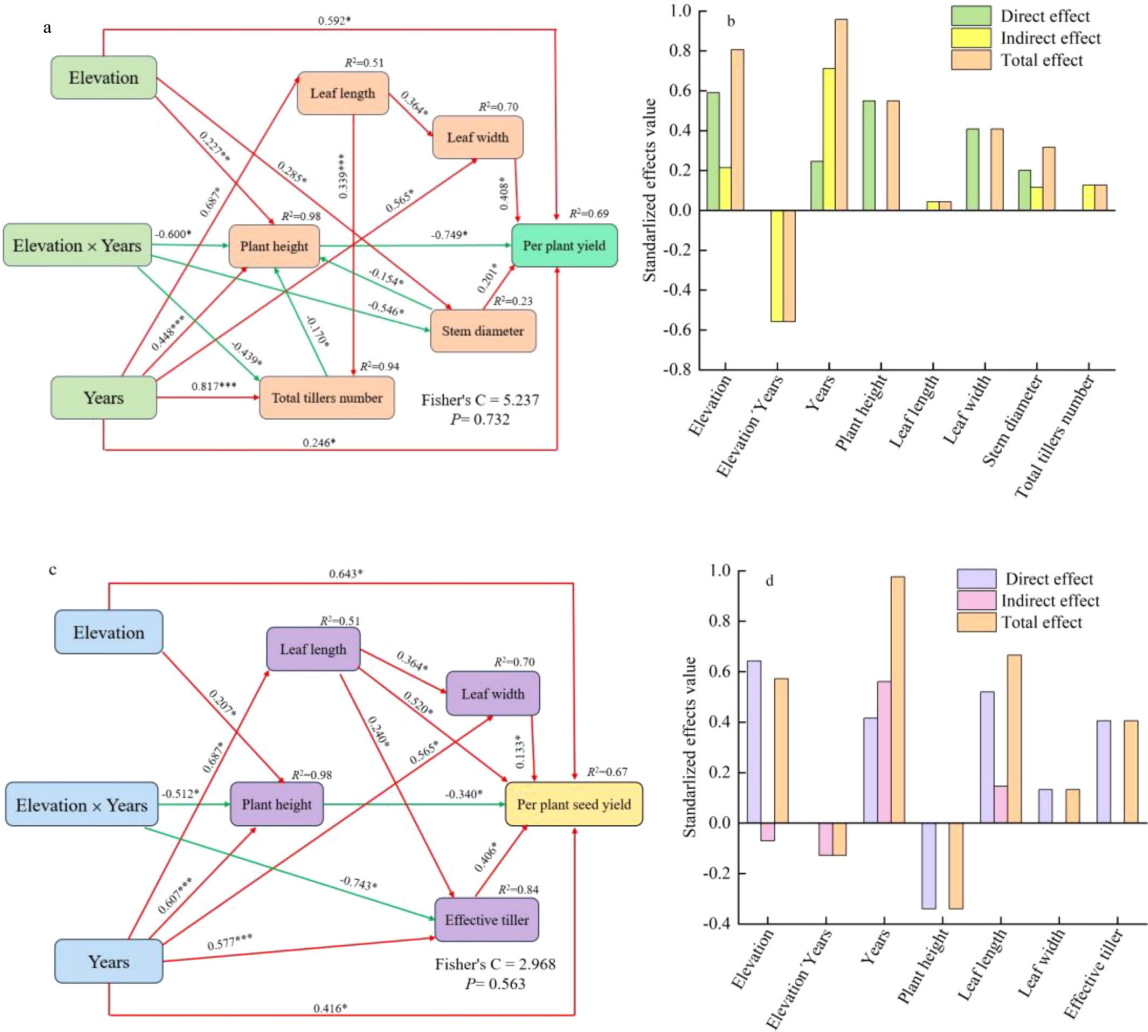
Structural equation modeling and the standardized effect values of various factors. **(a, c)** respectively depict the influence paths of elevation and years on per plant yield and seed yield, while **(b, d)** present the standardized effect values of various factors corresponding to the pathways for per plant yield and seed yield, respectively. Red arrows denote positive effect pathways, green arrows indicate negative effect pathways. Values represent standardized path coefficients. *, **, and *** denote significance at the 0.05, 0.01, and 0.001 confidence levels, respectively.

To elucidate the pathways and quantify the path coefficients through which elevation, year, and their interaction influence forage and seed yield, a structural equation model (SEM) was developed. The results demonstrated a good model fit (P = 0.732, Fisher’s C = 5.237). Elevation exerted an indirect effect on forage yield by influencing plant height and stem diameter. Year also had an indirect impact on forage yield through its effects on plant height, leaf length, leaf width, and total tiller number. The interaction between elevation and year indirectly influenced forage yield via plant height, stem diameter, and total tiller number. Leaf width and stem diameter had positive direct effects on forage yield, with path coefficients of 0.408 and 0.230, respectively. In contrast, plant height exhibited a significant negative direct effect, with a path coefficient of -0.749 (**Figure 7a**).

Elevation indirectly influenced seed yield by affecting plant height. Additionally, year had an indirect impact on seed yield through its effects on plant height, leaf length, leaf width, and the number of effective tillers. The interaction between elevation and year also exerted an indirect influence, mediated by plant height and the number of effective tillers. Leaf length, leaf width, and the number of effective tillers exhibited positive direct effects on seed yield, while plant height showed a negative direct effect. This model demonstrated a good fit (P = 0.563, Fisher’s C = 2.968) (**Figure 7c**).

Using the standardized path coefficients derived from the piecewise structural equation modeling (SEM), the contributions of each factor were quantified through effect decomposition. In the context of forage yield formation, plant height (total effect: -0.549) and leaf width (total effect: 0.408) emerged as the primary drivers. Path analysis indicated that elevation, year, and their interaction predominantly influenced forage yield by mediating these two traits (**Figure 7b**). Regarding seed yield formation, year, leaf length, and effective tiller number demonstrated the highest total effects, with values of 0.98, 0.67, and 0.41, respectively. Consequently, elevation and year primarily affected seed yield by modulating leaf length and effective tiller number (**Figure 7d**).

## 4 Discussion

Elevation serves as a critical environmental factor that influences plant growth and ecological adaptation on the Qinghai-Tibet Plateau (Ye et al., 2020). Changes in elevation are typically associated with gradients in ecological factors, including temperature, light, moisture, and soil nutrients, which profoundly impact plant morphogenesis, biomass accumulation, and reproductive strategies (Ma et al., 2010; Liu et al., 2024). This study conducted a two-year field observation of six wild *E. sibiricus* accessions sourced from varying elevations at a common experimental site, systematically elucidating the regulatory influence of the elevational gradient on the agronomic traits and production performance of *E. sibiricus*. Our findings revealed significant differences in key agronomic traits—such as plant height, stem diameter, leaf area, tiller number, forage yield, and seed yield—among *E. sibiricus* accessions from different elevations. Germplasms originating from higher elevations generally exhibited morphological characteristics such as plant dwarfing, reduced leaf size, and decreased tiller number, which likely reflect their inherent genetic background or adaptation history to their native habitats. These results are consistent with studies by Rahman et al. in the Himalayan region, which suggest that high-elevation environments, characterized by stressors such as low temperatures and intense UV radiation, induce plastic morphological responses in plants to optimize resource allocation and enhance stress resistance (Cho et al., 2018; Rahman et al., 2019). Accessions from higher elevations (e.g., 2019-004, 2019-005) exhibited suboptimal performance in certain traits, whereas those from mid-elevations (e.g., 2019-001, 2019-006) demonstrated superior performance across multiple agronomic indicators and yields. This observation suggests the presence of an optimal elevational range for *E. sibiricus*, beyond which its production performance may be compromised (Pan et al., 2023). Two-way ANOVA revealed that both elevation and year exerted highly significant effects on most traits, with their interaction notably influencing characteristics such as plant height, stem diameter, and tiller number. This finding emphasizes that the phenotypic expression of *E. sibiricus* arises from the interplay of genetic background and annual climatic conditions (Meyer et al., 2023; Sun et al., 2020). Consequently, it is essential to consider both genotype-by-environment interactions and their stability in the evaluation of germplasm and breeding efforts to improve regional adaptation and performance consistency.

Mantel correlation network analysis revealed significant positive correlations between both forage and seed yield and the agronomic traits of plant height, stem diameter, leaf length, and leaf width in *E. sibiricus*. This indicates synergistic effects among these traits during yield formation. Further analysis through piecewise structural equation modeling (SEM) demonstrated that forage yield is primarily influenced by plant height and leaf width. Functionally, plant height serves as a “spatial architect” and competitor, establishing the vertical structure necessary for light capture and providing the primary dry matter foundation for yield. In contrast, leaf width functions as an “energy engine,” directly facilitating organic synthesis by expanding the photosynthetic area and enhancing efficiency. The synergistic interaction between these traits collectively governs forage yield in *E. sibiricus* (Li et al., 2024; de Freitas et al., 2024). Seed yield formation depends on adequate photosynthetic products. Leaf length directly influences the photosynthetic area, supplying the material basis for yield, while the number of effective tillers determines the quantity of seed-bearing panicles—the fundamental units of yield—ultimately influencing seed harvest (Kamal et al., 2019; Zeng et al., 2024).These findings elucidate the ecological adaptation mechanisms of *E. sibiricus*, which enhances its production performance by modulating organ development and resource allocation strategies in response to varying elevational environments. K-means clustering categorized the six accessions into two distinct groups. The cluster from the 3000–4000 m elevation range (2019-001, 2019-002, 2019-003, and 2019-006) displayed superior comprehensive phenotypic traits, suggesting that this elevational range may represent the ecological optimum for *E. sibiricus.* The TOPSIS comprehensive evaluation further corroborated that accession 2019-001 (collected at 3147 m) excelled across multiple indicators and attained the highest similarity score, indicating robust ecological adaptability and high, stable yield potential. Consequently, accession 2019–001 should be prioritized as a candidate for breeding and ecological restoration of *E. sibiricus* in the eastern Qinghai-Tibet Plateau. However, this study has certain limitations, primarily related to the spatial dimension of the experimental design. Since all tested materials were evaluated at only a single elevation site, the conclusions drawn essentially reflect the performance of specific genotypes under a particular environmental condition. Therefore, future research should implement multi−environment trials by establishing test plots across different elevational gradients on the Qinghai–Tibet Plateau, using the elite accessions identified in this study along with appropriate controls. Such an approach would help to accurately delineate suitable promotion zones for superior germplasm and clarify their ecological adaptation limits.

Germplasms from mid-elevation regions (3000–4000 m) display enhanced overall production performance, indicating significant potential as elite genetic resources. This study offers a theoretical basis and material support for the ecological breeding of *E. sibiricus* in alpine regions. Future initiatives in germplasm innovation and promotion should focus on the exploration and utilization of accessions derived from this elevational range.

## 5 Conclusion

In comparison to the second year, third-year *E. sibiricus* demonstrated enhancements in both agronomic traits and yield. Wild *E. sibiricus* accessions were categorized into two distinct groups: one primarily found within the elevational range of 3000–4000 m and the other located above 4000 m. Forage yield was predominantly influenced by canopy architecture, which is determined by plant height and leaf width, while seed yield was influenced by the reproductive structure, supported by leaf length and effective tillering. Accession 2019–001 attained the highest comprehensive evaluation score and exhibited optimal performance, indicating that its native elevation of 3147 m may represent a suitable elevational gradient for wild *E. sibiricus*. This finding offers valuable insights for the breeding of *E. sibiricus* in alpine regions.

## Data Availability

The datasets presented in this study can be found in online repositories. The names of the repository/repositories and accession number(s) can be found in the article/supplementary material.
